# Reporting of Study Participant Demographic Characteristics and Demographic Representation in Premarketing and Postmarketing Studies of Novel Cancer Therapeutics

**DOI:** 10.1001/jamanetworkopen.2021.7063

**Published:** 2021-04-20

**Authors:** Tanvee Varma, Joshua D. Wallach, Jennifer E. Miller, Dominic Schnabel, Joshua J. Skydel, Audrey D. Zhang, Michaela A. Dinan, Joseph S. Ross, Cary P. Gross

**Affiliations:** 1 Yale School of Medicine, New Haven, Connecticut; 2 Department of Environmental Health Sciences, Yale School of Public Health, New Haven, Connecticut; 3 Department of Internal Medicine, Yale School of Medicine, New Haven, Connecticut; 4 Bioethics International, New York, New York; 5 Harvard Medical School, Boston, Massachusetts; 6 Tufts University School of Medicine, Boston, Massachusetts; 7 Department of Medicine, Duke University School of Medicine, Durham, North Carolina; 8 Department of Population Health Sciences, Duke University, Durham, North Carolina; 9 Duke Cancer Institute, Durham, North Carolina; 10 Section of General Internal Medicine, Department of Internal Medicine, Yale School of Medicine, New Haven, Connecticut; 11 Department of Health Policy and Management, Yale School of Public Health, New Haven, Connecticut; 12 Center for Outcomes Research and Evaluation, Yale New Haven Hospital, New Haven, Connecticut; 13 Cancer Outcomes, Public Policy, and Effectiveness Research (COPPER) Center, Yale University, New Haven, Connecticut; 14 Department of Chronic Disease Epidemiology, Yale School of Public Health, New Haven, Connecticut

## Abstract

**Importance:**

Adequate representation of demographic subgroups in premarketing and postmarketing clinical studies is necessary for understanding the safety and efficacy associated with novel cancer therapeutics.

**Objective:**

To characterize and compare the reporting of demographic data and the representation of individuals by sex, age, and race in premarketing and postmarketing studies used by the Food and Drug Administration (FDA) to evaluate novel cancer therapeutics.

**Design, Setting, and Participants:**

In this cross-sectional study, premarketing and postmarketing studies for novel cancer therapeutics approved by the FDA from 2012 through 2016 were identified. Study demographic information was abstracted from publicly available sources, and US cancer population demographic data was abstracted from US Cancer Statistics. Analyses were conducted from February 25 through September 21, 2020.

**Main Outcomes and Measures:**

The percentages of trials reporting sex, age, and race/ethnicity were calculated, and participation to prevalence ratios (PPRs) were calculated by dividing the percentage of study participants in each demographic group by the percentage of the US cancer population in each group. PPRs were constructed for premarketing and postmarketing studies and by cancer type. Underrepresentation was defined as PPR less than 0.8.

**Results:**

From 2012 through 2016, the FDA approved 45 cancer therapeutics. The study sample included 77 premarketing studies and 56 postmarketing studies. Postmarketing studies, compared with premarketing studies, were less likely to report patient sex (42 studies reporting [75.0%] vs 77 studies reporting [100%]; *P* < .001) and race (27 studies reporting [48.2%] vs 62 studies reporting [80.5%]; *P* < .001). Women were adequately represented in premarketing studies (mean [SD] PPR, 0.91; 95% CI, 0.90-0.91) and postmarketing studies (mean PPR, 1.00; 95% CI, 1.00-1.01). Although older adults and Black patients were underrepresented in premarketing studies (older adults: mean PPR, 0.73; 95% CI, 0.72-0.74; Black patients: mean PPR, 0.32; 95% CI, 0.31-0.32), these groups continued to be underrepresented in postmarketing studies (older adults: mean PPR, 0.75; 95% CI, 0.75-0.76; Black patients: mean PPR, 0.21; 95% CI, 0.21-0.21).

**Conclusions and Relevance:**

This study found that older adults and Black patients were underrepresented in postmarketing studies of novel cancer therapeutics to a similar degree that they were underrepresented in premarketing studies. These findings suggest that postmarketing studies are not associated with improvements to gaps in demographic representation present at the time of FDA approval.

## Key Points

**Question:**

Are the demographic characteristics of patients enrolled in premarketing and postmarketing studies used by the US Food and Drug Administration (FDA) to evaluate the safety and efficacy of novel cancer therapeutics clearly reported and representative of the US cancer population?

**Findings:**

In this cross-sectional study of 77 premarketing studies and 56 postmarketing studies for FDA-approved cancer therapeutics, there was inconsistent reporting of demographic data and underrepresentation of older adults and Black patients in premarketing and postmarketing studies of cancer therapeutics.

**Meaning:**

Premarketing and postmarketing studies used by the FDA to evaluate novel cancer therapeutics may not adequately represent the US cancer population.

## Teaser

This cross-sectional study investigates demographic data reporting and demographic representation by sex, age, and race/ethnicity in premarketing and postmarketing studies of novel cancer therapeutics.

## Introduction

In the United States, the Food and Drug Administration (FDA) is responsible for the approval of novel therapeutics through the evaluation of clinical trials.^1^ Clinical trials are done to ensure that a therapeutic is safe and efficacious in the population expected to use the therapeutic. Therefore, clinical trial participants should be representative of the patient population.^2^ However, there are well-documented concerns that women, older adults, and racial/ethnic minority groups are underrepresented in premarketing studies for cancer therapeutics.^3,4,5,6^ Additionally, some evidence suggests that race/ethnicity data are underreported in cancer clinical trials.^3^

Accordingly, the FDA has had increased focus on improving demographic representation in clinical trials.^7,8^ The FDA presented an action plan in 2014 to improve demographic subgroup analysis in the evaluation of new therapeutics, with specific guidelines for improving the quality of demographic data collected in postmarketing surveillance systems.^9^

Postmarketing studies with diverse patient populations are especially important for understanding the safety and efficacy profile of cancer therapeutics, given that many cancer therapeutics are approved for marketing at earlier stages with less robust evidence through expedited approval pathways; for example, 76% of cancer therapeutics received priority review over that last 3 decades.^10,11,12^ Because postmarketing studies are meant to address any uncertainties about safety and efficacy at the time of approval and address the well-known underrepresentation of demographic subgroups in premarketing studies, adequate demographic representation in postmarketing studies is particularly important. The FDA has asserted that a more diverse patient population in postmarketing studies will “provide valuable information to better inform providers and the FDA about the safe and effective use of new therapeutics.”^13^

There are some concerns with postmarketing requirements (PMRs) and postmarketing commitments (PMCs), including that many are not associated with improvements to gaps in the foundation of clinical evidence present at the time of FDA approval.^14,15,16^ However, to our knowledge, there is no evidence evaluating whether postmarketing studies for cancer therapeutics are associated with improved representation of women, older adults, or racial/ethnic minority groups, compared with premarketing studies. To address these knowledge gaps, we characterized and compared the quality of demographic data reporting and demographic representation of patients enrolled in premarketing studies vs postmarketing studies evaluating novel cancer therapeutics approved by the FDA between 2012 and 2016.

## Methods

The Yale Human Investigations Committee office determined that this cross-sectional study was not subject to institutional review board approval or informed consent because the study was conducted using only publicly available study reports and manuscripts. In this study, we identified all novel therapeutics approved by the FDA for an oncologic indication from 2012 through 2016. Using FDA medical review documents, we identified premarketing and postmarketing studies for these therapeutics. Using medical review documents, ClinicalTrials.gov, journal publications, and sponsors’ websites, we analyzed reporting of patient demographic data and compared the demographic composition of these studies with the demographic composition of the 2012 to 2016 US cancer population by indication.

### Study Sample

Using the yearly published Center for Drug Evaluation and Research New Molecular Entity Drug and Original Biologic Approvals list, 2 investigators (TV and JJS) identified all novel drugs and biologics approved by the FDA for oncologic indications from 2012 through 2016.^17^ To ensure that there was enough time for the completion and reporting of results of postmarketing studies, cancer therapeutics approved after 2016 were not included in our sample. Using medical review documents in the Drugs@FDA database,^18^ we determined whether therapeutics were approved through expedited pathways (ie, priority review, accelerated approval, breakthrough designation, and fast track) or designated as orphan drugs, as well as each therapeutic’s indication according to cancer type. From the Review Strategy section of the medical review documents, we identified all clinical studies that provided some evidence regarding the efficacy of the therapeutic. Two investigators (TV and DS) classified studies as *pivotal* if they were explicitly described as pivotal or if the studies provided evidence of efficacy or safety that was essential to FDA approval, as noted in the medical review documents. All other trials were classified as *nonpivotal*. Two investigators (JJS and JDW) identified all PMRs and PMCs outlined in the original approval letters available in the Drugs@FDA database.^14,15,16^

Among this sample of premarketing and postmarketing studies, we excluded studies that evaluated only the therapeutics’ safety, pharmacokinetics and pharmacodynamics, bioavailability, dose escalation, or drug interactions (eTable 1 in the Supplement). Additionally, Phase 1 studies, extension studies, expanded access studies, and studies that included patients with a different indication or only healthy patients were excluded. All PMRs and PMCs that were nonclinical studies, extension studies, recruiting patients as of July 2020, aggregates of unspecified trials, or for trials listed in more than 1 PMR or PMC were excluded (eTable 1 in the Supplement). Trials that were designated as both premarketing studies (pivotal or nonpivotal) and postmarketing studies (PMR or PMC) were included in our sample only as postmarketing studies.

### Demographic Characteristics of Study Patients

To determine the demographic characteristics of patients enrolled in our sample of premarketing studies, we abstracted the number of participants who were women, were older adults (defined as ages 65 years or older), and belonged to racial/ethnic minority groups from medical review documents in the Drugs@FDA database. If demographic information was not available in these documents, we abstracted data from ClinicalTrials.gov, indexed publications from the clinical trial entry on ClinicalTrials.gov, or clinical trials synopses on the study sponsor’s website, in this order. Demographic information in the FDA’s medical review documents, even if available for only a portion of the study sample, superseded demographic information of the entire trial in ClinicalTrials.gov.

For postmarketing studies, we abstracted demographic information from ClinicalTrials.gov, indexed publications of the clinical trial entry on ClinicalTrials.gov, or clinical trials synopses on the study sponsor’s website. Given that many PMRs and PMCs did not have results reported on ClinicalTrials.gov, we also searched the Scopus database for publications (Elsevier) using a previously described approach.^14,15,19^

Data on the demographic distribution of patients diagnosed with cancer in the United States was abstracted from the 2012 to 2016 US Cancer Statistics data set, which includes cancer registry data from the National Program of Cancer Registries and the National Cancer Institute’s Surveillance, Epidemiology, and End Results (SEER) data set.^20^ This data set includes demographic data for the proportion of US cancer patients who identify as women; older adults; and White, Black, or Asian, by cancer type. Analyses were conducted from February 25 through September 21, 2020.

### Statistical Analysis

We calculated the percentage of premarketing and postmarketing studies that reported the proportion of patients who were women and older adults and the proportion by race/ethnicity. To assess whether there was a significant difference in demographic data reporting between premarketing and postmarketing studies, we calculated a χ^2^ test of independence. Results were considered statistically significant at a *P* value of < .05. All tests were 2-tailed. Data analyses were conducted from February 25 through September 21, 2020, using Excel version 16.16.27 (Microsoft Corporation).

We aggregated demographic data by cancer type and calculated the proportion of patients who were women and older adults and the proportion by race (ie, White, Black, or Asian). To evaluate the demographic composition of patients in trials relative to the disease population, we constructed the participation to prevalence ratio (PPR), as developed by Poon et al.^21^ The PPR was calculated by dividing the percentage of a particular population among the study participants (eg, percentage of women among participants in lung cancer trials) by the percentage of the particular population in the disease population (eg, percentage of women among individuals in the US with lung cancer) by cancer type. A PPR from 0.8 to 1.2 indicates adequate representation; a PPR less than 0.8 indicates underrepresentation, and a PPR greater than 1.2 indicates overrepresentation.^21^ Within each indication, we compared the PPR of premarketing and postmarketing studies by sex, age, and race. We constructed CIs using the CI formula for sample proportions using the total number of study participants for each indication as the denominator.^22^ Only studies with complete race data were included in the calculation of PPR by race

We were unable to calculate the PPR for basal cell carcinoma or for Asian patients with neuroblastoma because the data were unavailable in US Cancer Statistics. Age data for the population diagnosed with soft tissue sarcoma were taken from SEER.^23^ We did not calculate the PPR for older adults with neuroblastoma because the incidence of neuroblastoma among older adults is very low.^24^

## Results

Among 45 therapeutics approved by the FDA for an oncologic indication from 2012 through 2016, 31 were drugs and 14 were biologics (Table); 24 therapeutics (53.3%) received accelerated approval, and 35 therapeutics (77.8%) were granted orphan drug status. The therapeutics in our sample were approved for 16 broad indications (eTable 2 in the Supplement). Multiple myeloma and leukemia were the most common indications (Table). We identified 77 premarketing studies (including 42 pivotal studies [54.5%] and 35 nonpivotal studies [45.5%]) and 56 postmarketing studies (described in 45 PMRs [80.4%] and 11 PMCs [19.6%]) for the therapeutics in our sample.

**Table.** Characteristics of Oncologic Therapeutic Approvals

Characteristic	No. (%) (N = 45)
Type	
Drug	31 (68.9)
Biologic	14 (31.1)
Year	
2012	11 (24.4)
2013	8 (17.8)
2014	8 (17.8)
2015	14 (31.1)
2016	4 (8.9)
Approval pathway	
Priority review	34 (75.6)
Accelerated approval	24 (53.3)
Fast track	24 (53.3)
Breakthrough	18 (40.0)
Orphan drug status	35 (77.8)
Indication	
Multiple myeloma	6 (13.3)
Leukemia	6 (13.3)
Melanoma	5 (11.1)
Lung cancer	5 (11.1)
Breast cancer	3 (6.7)
Colorectal cancer	3 (6.7)
Non-Hodgkin lymphoma	3 (6.7)
Ovarian cancer	2 (4.4)
Thyroid cancer	2 (4.4)
Basal cell carcinoma	2 (4.4)
Prostate cancer	2 (4.4)
Soft tissue sarcoma (including liposarcoma)	2 (4.4)
Other^a^	4 (8.8)

^a^ The indications for the 4 therapeutics are urothelial cancer, neuroblastoma, gastric cancer, and renal cell carcinoma.

### Availability of Demographic Data

**Figure 1.**
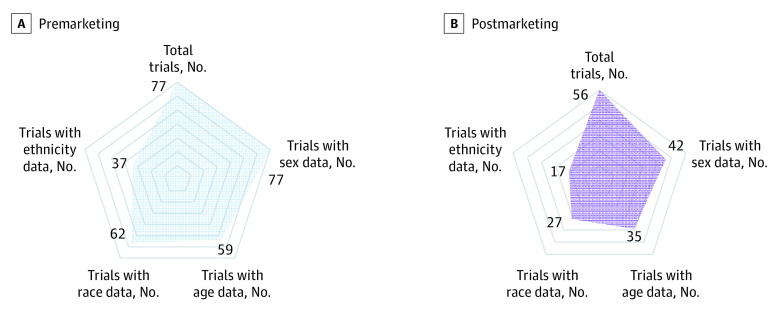
Availability of Demographic Data

Postmarketing studies, compared with premarketing studies, were less likely to report on patient sex (42 studies reporting [75.0%] vs 77 studies reporting [100%]; *P* < .001) and race (27 studies reporting [48.2%] vs 62 studies reporting [80.5%]; *P* < .001) (Figure 1). There was no significant difference between postmarketing and premarketing studies in reporting of the proportion of older adults (35 studies [62.5%] vs 59 studies [76.6%]; *P* = .08) or the reporting of ethnicity (17 studies [30.4%] vs 37 studies [48.1%]; *P* = .07). We found that while all premarketing studies reported some demographic data, only 42 postmarketing studies [75.0%] reported some demographic data (eTable 3 in the Supplement).

### Demographic Representation in Premarketing and Postmarketing Studies

#### Sex

**Figure 2.**
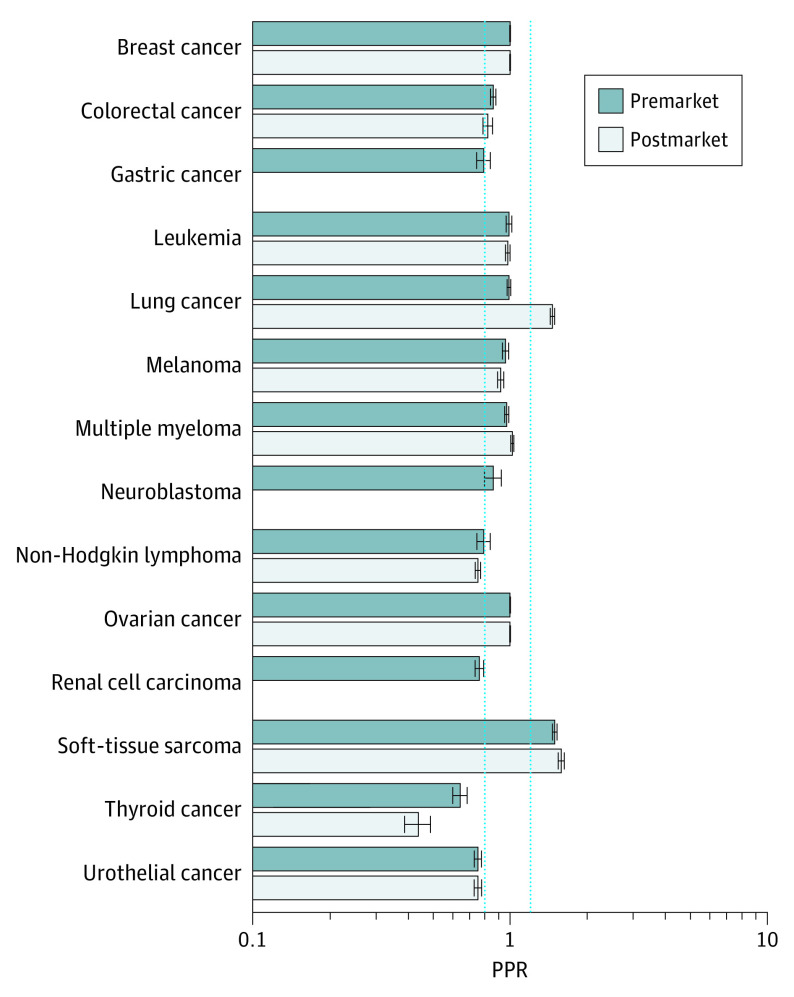
Participation to Prevalence Ratio (PPR) for Women The PPR is calculated by dividing the proportion of study patients who are women by the proportion of US cancer patients who are women, by cancer type. A PPR from 0.8 to 1.2 indicates adequate representation, a PPR less than 0.8 indicates underrepresentation, and a PPR greater than 1.2 indicates overrepresentation. Dotted blue lines indicate these boundaries; error bars, CIs. The number of trials included in the PPR by cancer type can be found in eTable 3 in the Supplement. Data for sex were missing for 14 postmarketing studies.

Women were adequately represented in premarketing studies (mean PPR, 0.91; 95% CI, 0.90-0.91) and postmarketing studies (mean PPR, 1.00; 95% CI, 1.00-1.01) across all therapeutics (Figure 2). Of 3 indications in which women were underrepresented in premarketing studies (ie, PPR < 0.8) (renal cell carcinoma: 0.76; 95% CI, 0.73-0.79; thyroid cancer: 0.64; 95% CI, 0.60-0.68; and urothelial cancer: 0.75; 95% CI, 0.72-0.77), women continued to be underrepresented in postmarketing studies for all indications except renal cell carcinoma, for which there were no postmarketing studies (thyroid cancer: 0.44; 95% CI, 0.39-0.49 and urothelial cancer: 0.75; 95% CI, 0.73-0.79) (Figure 2). Study-specific PPRs for women by cancer type can be found in eFigure 1 in the Supplement.

#### Age

**Figure 3.**
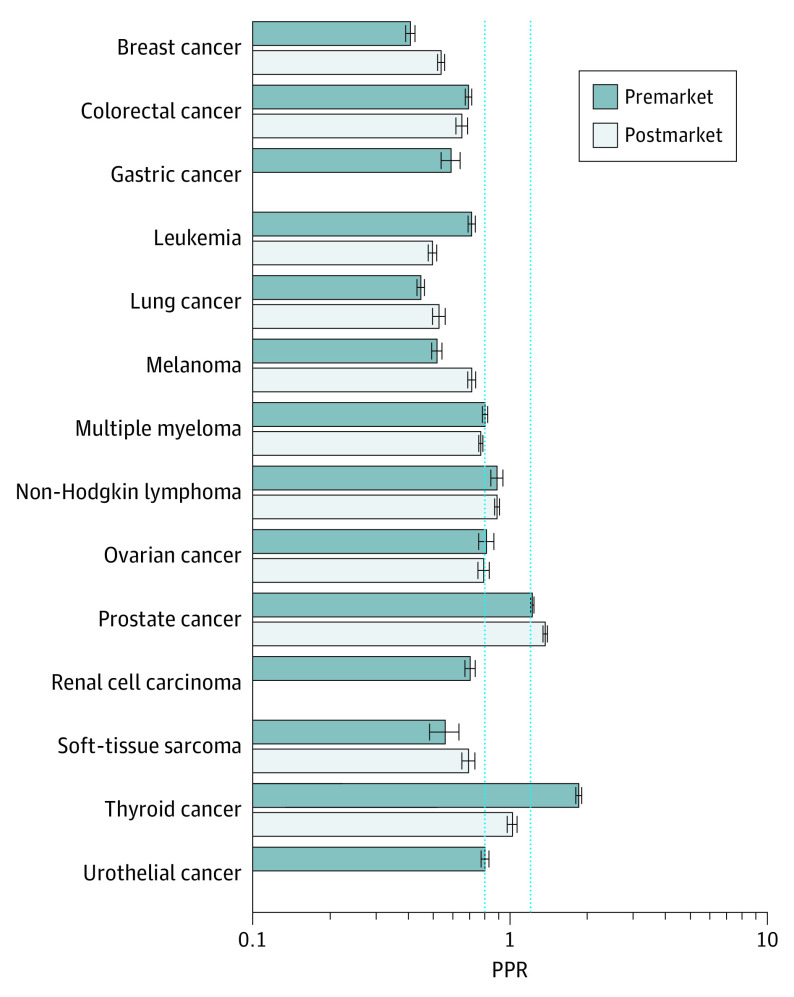
Participation to Prevalence Ratio (PPR) for Older Adults (Aged ≥65 Years) The PPR is calculated by dividing the proportion of study patients who are older adults by the proportion of US cancer patients who are older adults, by cancer type. A PPR from 0.8 to 1.2 indicates adequate representation, a PPR less than 0.8 indicates underrepresentation, and a PPR greater than 1.2 indicates overrepresentation. Dotted blue lines indicate these boundaries; error bars, CIs. The number of trials included in the PPR by cancer type can be found in eTable 3 in the Supplement. Data on older adults were missing for 18 premarketing studies and 21 postmarketing studies.

Older adults were underrepresented in premarketing studies (mean PPR, 0.73; 95% CI, 0.72-0.74) and postmarketing studies (mean PPR, 0.75; 95% CI, 0.75-0.76). There were 8 indications in which older adults were underrepresented in premarketing studies according to PPR: breast cancer: 0.41 (95% CI, 0.49-0.43); colorectal cancer: 0.69 (95% CI, 0.67-0.71); gastric cancer: 0.59 (95% CI, 0.54-0.64); leukemia: 0.71 (95% CI, 0.69-0.74); lung cancer: 0.45 (95% CI, 0.43-0.46); melanoma: 0.52 (95% CI, 0.49-0.54); renal cell carcinoma: 0.70 (95% CI, 0.66-0.73); and soft tissue sarcoma: 0.56 (95% CI, 0.49-0.63). Among these, 6 indications had postmarketing studies that reported patient age (Figure 3). These 6 indications continued to have underrepresentation of older adults in postmarketing studies according to PPR: breast cancer: 0.54 (95% CI, 0.52-0.55); colorectal cancer: 0.65 (95% CI, 0.62-0.69); leukemia: 0.45 (95% CI, 0.48-0.52); lung cancer: 0.53 (95% CI, 0.50-0.56); melanoma: 0.71 (95% CI, 0.68-0.73); and soft tissue sarcoma: 0.69 (95% CI, 0.65-0.73). Study-specific PPRs for older adults by cancer type can be found in eFigure 2 in the Supplement.

The absolute difference in the proportion of older adults in our sample of studies and the proportion in the US cancer population by indication ranged from 156 of 560 individuals (39.8%) vs 52 243 of 243 109 individuals (21.5%), for a difference of 18.3 percentage points, for thyroid cancer to 1287 of 3947 patients (30.9%) vs 762 725 of 1 104 350 individuals (69.1%), for a difference of −38.2 percentage points, for lung cancer in premarketing studies. It ranged from 690 of 858 individuals (80.4%) vs 555 830 of 946 936 individuals (58.7%) , for a difference of 21.7 percentage points, for prostate cancer to 364 of 1114 individuals (36.6%) vs 762 725 of 1 104 350 individuals (69.1%), for a difference of −32.5 percentage points, for lung cancer in postmarketing studies (eTable 4 in the Supplement).

#### Race

**Figure 4.**
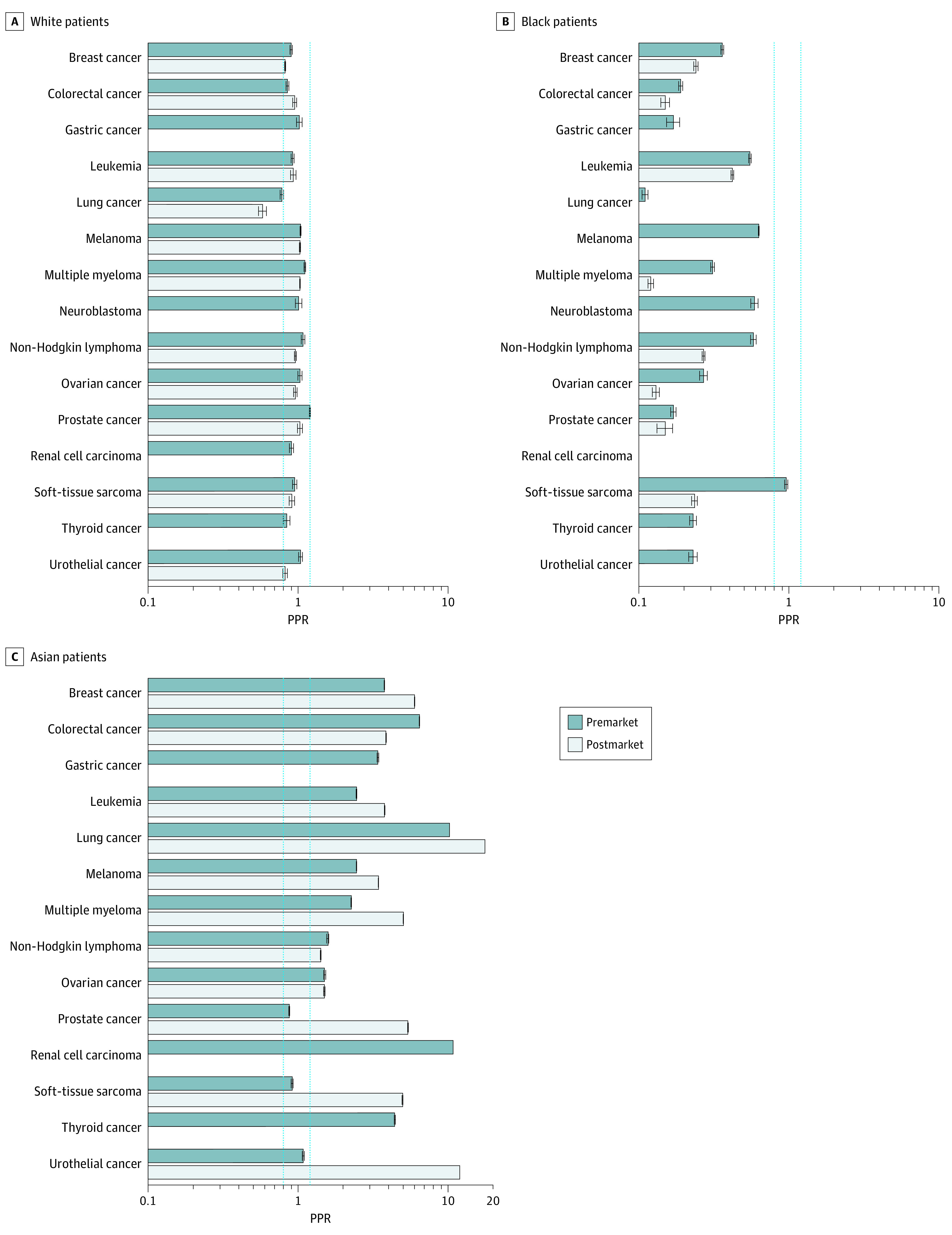
Participation to Prevalence Ratio (PPR) by Race The PPR is calculated by dividing the proportion of study patients who are White, Black, or Asian by the proportion of US cancer patients who are White, Black, or Asian, by cancer type. A PPR from 0.8 to 1.2 indicates adequate representation, a PPR less than 0.8 indicates underrepresentation, and a PPR greater than 1.2 indicates overrepresentation. Dotted blue lines indicate these boundaries; error bars, CIs. The number of trials included in the PPR by cancer type can be found in eTable 3 in the Supplement. Data on race were missing for 15 premarketing studies and 29 postmarketing studies.

White patients were adequately represented in premarketing studies (mean PPR, 0.95; 95% CI, 0.94-0.85) and postmarketing studies (mean PPR, 0.92; 95% CI, 0.91-0.93) (Figure 4). Black patients were underrepresented in premarketing studies (mean PPR, 0.32; 95% CI, 0.31-0.32) and postmarketing studies (mean PPR, 0.21; 95% CI, 0.20-0.22). Among 15 indications that had race data, Black patients were underrepresented in premarketing studies for 14 indications according to PPR: breast cancer 0.32 (95% CI, 0.31-0.33); colorectal cancer: 0.19 (95% CI, 0.19-0.20); gastric cancer: 0.17 (95% CI, 0.15-0.19); leukemia: 0.55 (95% CI, 0.53-0.56); lung cancer: 0.11 (95% CI, 0.10-0.11); melanoma: 0.63 (95% CI, 0.63-0.63); multiple myeloma: 0.31 (95% CI, 0.30-0.32); neuroblastoma: 0.59 (95% CI, 0.55-0.62); non-Hodgkin lymphoma: 0.58 (95% CI, 0.56-0.61); ovarian cancer: 0.27 (95% CI, 0.26-0.29); prostate cancer: 0.17 (95% CI, 0.16-0.17); renal cell carcinoma: 0.05 (95% CI, 0.04-0.05); thyroid cancer: 0.23 (95% CI, 0.22-0.24); and urothelial cancer 0.23 (95% CI, 0.21-0.24) (Figure 4). Of these 14 indications, 10 indications had postmarketing studies that reported patient race. All 10 indications continued to have underrepresentation of Black patients in postmarketing studies according to PPR: breast cancer: 0.24 (95% CI, 0.23-0.25); colorectal cancer: 0.15 (95% CI, 0.14-0.16); leukemia: 0.42 (95% CI, 0.41-0.43); lung cancer: 0.07 (95% CI, 0.07-0.08); melanoma: 0; multiple myeloma: 0.12 (95% CI, 0.11-0.12); non-Hodgkin lymphoma: 0.31 (95% CI, 0.30-0.32); ovarian cancer: 0.13 (95% CI, 0.12-0.14); prostate cancer: 0.20 (95% CI, 0.19-0.22); soft tissue sarcoma: 0.23 (95% CI, 0.22-0.25); urothelial cancer: 0.06 (95% CI, 0.06-0.07).

The absolute difference in the proportion of Black individuals in our sample of studies and the proportion in the US cancer population by indication ranged from 2 of 662 individuals (0.30%) vs 1875 of 391 393 individuals (0.48%), for a difference of −0.18 percentage points, for melanoma to 164 of 2573 individuals (6.4%) vs 26 077 of 127 402 individuals (20.5%), for a difference of −14.1 percentage points, for multiple myeloma in premarketing studies. It ranged from 0 of 945 individuals (0%) vs 1875 of 371 542 individuals (0.48%), for a difference of −0.48 percentage points, for melanoma to 58 of 2278 individuals (2.6%) vs 26 077 of 95 670 individuals (20.8%), for a difference of −18.2 percentage points for multiple myeloma in postmarketing studies (eTable 4 in the Supplement).

Asian patients were overrepresented in premarketing studies (mean PPR, 3.99; 95% CI, 3.99-4.00) and postmarketing studies (mean PPR, 4.26; 95% CI, 4.26-4.27). In premarketing studies, Asian patients were overrepresented for 11 indications according to PPR: breast cancer: 3.27 (95% CI, 3.26-3.29); colorectal cancer: 6.40 (95% CI, 6.38-6.42); gastric cancer: 3.39 (95% CI, 3.35-3.44); leukemia: 2.44 (95% CI, 2.43-2.46); lung cancer: 10.16 (95% CI, 10.14-10.18); melanoma: 2.44 (95% CI, 2.43-2.44); multiple myeloma: 2.25 (95% CI, 2.24-2.25); non-Hodgkin lymphoma: 1.57 (95% CI, 1.54-1.59); ovarian cancer: 1.50 (95% CI, 1.48-1.53); renal cell carcinoma: 10.72 (95% CI, 10.69-10.74); and thyroid cancer: 4.39 (95% CI, 4.35-4.43) (Figure 4). Of these 11 indications, 8 indications had postmarketing studies that reported patient race. Asian patients were overrepresented in postmarketing studies for all 8 indications according to PPR: breast cancer: 5.95 (95% CI, 5.94-5.96); colorectal cancer: 3.84 (95% CI, 3.83-3.86); leukemia: 3.76 (95% CI, 3.75-3.78); lung cancer: 17.47 (95% CI, 17.43-17.51); melanoma: 3.41 (95% CI, 3.41-3.42); multiple myeloma: 5.00 (95% CI, 4.99-5.01); non-Hodgkin lymphoma: 1.41 (95% CI, 1.41-1.51); ovarian cancer 1.49 (95% CI, 1.48-1.51); prostate cancer: 5.37 (95% CI, 5.33-5.40); soft tissue sarcoma: 4.94 (95% CI, 4.90-4.97); and urothelial cancer: 10.01 (95% CI, 9.98-10.04). Study-specific PPRs by race and by cancer type can be found in eFigure 3 in the Supplement.

## Discussion

In this cross-sectional study of 45 cancer therapeutics, we found that postmarketing studies were less likely to report patient sex and race than premarketing studies. We found little evidence that postmarketing studies were associated with improvements to poor demographic representation in premarketing studies. Older adults and Black patients were consistently underrepresented in premarketing and postmarketing studies. Inadequate demographic representation in premarketing and postmarketing studies calls into question the generalizability of cancer clinical trial results to clinical practice as well as the FDA’s ability to encourage or enforce demographic representation in clinical studies.

Older adults were underrepresented in premarketing and postmarketing studies for most cancer indications, suggesting that postmarketing studies are not associated with improvements to the representation of older adults at the time of approval. Given that cancer incidence increases with age, representation of older adults is particularly important.^25^ There may also be differences in drug response and toxic effects by age.^26^ Additionally, older adults are more likely to have comorbidities and use medications concomitantly, both of which can be associated with therapeutics’ safety and efficacy outcomes.^27^ In fact, comorbid conditions and concomitant medications are often exclusion criteria in cancer clinical trials.^28,29,30^ While the FDA has provided guidelines for increasing enrollment of older adults, our findings suggest that there are further opportunities to improve representation of older adults in cancer clinical trials.^31^

Our study suggests that Black patients are significantly underrepresented in premarketing and postmarketing studies for cancer therapeutics. Prior work suggests that Black patients are underrepresented in industry-sponsored and federally sponsored cancer clinical trials, although there is some evidence suggesting that representation is improving in federally sponsored trials.^3,5,6,32,33^ It is worth noting that all trials in our sample were industry-sponsored. Barriers to representation of Black patients in cancer clinical trials include structural barriers (eg, transportation, child care, and access to health care) and a disproportionate burden of comorbidities.^34,35,36,37,38,39,40^ Furthermore, there is a well-documented and justified mistrust of research institutions among Black patients stemming from a long history of exploitation of Black patients by medical research.^41^ Structural racism in the US health care system continues to reinforce this mistrust.^42^ Studies of interpersonal discrimination have found that research and clinical professionals view Black patients as less likely to enroll in clinical research and, therefore, clinicians spend less time discussing clinical trials with Black patients.^43,44,45^ It is particularly problematic that Black patients are underrepresented in postmarketing studies, given that postmarketing studies evaluate therapeutics for which there is already preliminary evidence suggesting effectiveness. It is worth noting that representation of racial/ethnic minority groups in clinical trials is important not because there is a biological basis for race but because understanding the safety and efficacy of novel therapeutics requires diverse patient populations.

We found that women were adequately represented in premarketing and postmarketing studies. Our findings support current literature that suggests that women are adequately represented in cancer clinical trials.^5,6,46^ Interestingly, Asian patients were overrepresented in premarketing and postmarketing studies. This may be because several studies in our sample were done in Asian countries. Although our findings do mirror current evidence that Asian patients are adequately represented or overrepresented in clinical trials, there is evidence of barriers to clinical trial participation for Asian patients.^3,47,48^

The FDA published several guidelines from 2014 to 2020 for improving reporting and participation of women, older patients, and racial and ethnic minority groups in clinical research.^8,9,49^ However, our findings suggest that translating these guidelines into practice remains a challenge. Several strategies have been proposed to increase the inclusion of older adults and Black patients in cancer clinical trials. In February 2020, the FDA and the American Association of Cancer Research made recommendations to improve Black patient representation in multiple myeloma clinical trials, including designing protocols with explicit targets for representation of racial/ethnic minority groups and specific plans for how these targets will be met.^50^ Another strategy is to create incentives for manufacturers to test therapeutics in older adults by providing manufacturers with 6-month patent extensions.^51,52^ This would be similar to the pediatric market exclusivity incentive, which has been associated with increased research among pediatric patients.^53^

Given that exclusion criteria based on comorbidities or concomitant medication use can disproportionately exclude older adults and Black patients, these criteria should be included only when necessary.^54,55^ Addressing logistical barriers of clinical trial participation, as well as developing partnerships with community organizations, may also increase participation.^2,56,57^ Ultimately, the FDA should require demographic representation in premarketing and postmarketing studies that approximates the population expected to use the therapeutic by investing in adequate recruitment a priori to ensure that ensuring demographic representation does not risk delaying study completion for new drugs.

### Limitations

This study has a number of limitations. First, several studies were missing demographic data, which undermined our ability to evaluate demographic representation across all trials in our sample. For instance, we were unable to calculate the PPR for Latinx patients. This speaks to the inconsistency of race/ethnicity subgroup reporting in cancer clinical trials, as reported in other studies.^3,58^ Second, we did not exclude trials conducted outside of the United States, and therefore, not all trial populations in this study demographically represent the US population. This is particularly true for the PPR for Asian patients, given that several premarketing studies were done in Japan. We justify their inclusion in this analysis because these studies were submitted by sponsors to support approval of the therapeutic in the United States. Third, our study did not represent clinical studies for recently approved therapeutics. We selected therapeutics approved from 2012 through 2016 to allow for adequate completion and results reported from postmarketing studies. Fourth, our analysis of demographic data reporting in premarketing and postmarketing studies had a relatively small sample size. We found that there was no significant difference between premarketing and postmarketing studies in reporting of age or ethnicity, despite consistent trends that postmarketing studies reported less demographic data than premarketing studies. Fifth, our study did not consider any genetic factors that may contribute to the racial composition of trial participants. For example, research suggests that the *EGFR* mutation may be more prevalent in people of East Asian ancestry, which may be associated with the disproportionately high representation of Asian individuals in some lung cancer studies.^59,60^

## Conclusions

In this study, we characterized the representation of individuals by sex, age, and race in 77 premarketing studies and 56 postmarketing studies for 45 novel cancer therapeutics approved by the FDA from 2012 through 2016. We found that postmarketing studies were more likely to underreport demographic data about patient sex and race compared with premarketing studies. Additionally, demographic representation in postmarketing studies was not different from that of premarketing studies, in which older adults and Black patients were significantly underrepresented. Ultimately, these findings suggest that the FDA must do more to improve the transparency of clinical trial demographic data and to ensure that women, older adults, and racial/ethnic minority groups are adequately represented in premarketing and postmarketing studies of novel cancer therapeutics.

## Notes

Supplement. **eTable 1.** Inclusion and Exclusion Criteria **eTable 2.** Novel Therapeutics With Oncologic Approvals by Cancer Type (2012-2016) **eTable 3.** Availability of Demographic Data by Cancer Type **eTable 4.** Absolute Difference in Proportion of Women and Older Adults and Proportion by Race Between Study Sample and Patient Population by Cancer Type **eFigure 1.** Participation to Prevalence Ratio (PPR) for Women by Cancer Type **eFigure 2.** Participation to Prevalence Ratio (PPR) for Older Adults by Cancer Type **eFigure 3.** Participation to Prevalence Ratio (PPR) for Race by Cancer Type
