# Error in Author Degrees

**DOI:** 10.1001/jamanetworkopen.2021.14399

**Published:** 2021-05-18

In the Original Investigation titled “Reporting of Study Participant Demographic Characteristics and Demographic Representation in Premarketing and Postmarketing Studies of Novel Cancer Therapeutics,”^1^ published April 20, 2021, there were errors in the author degrees listed. Mr Schnabel's degree should have been MPH, and Mr Skydel's degree should have been BS. This article has been corrected.^1^
