# Supplementary material for: Reporting of Study Participant Demographic Characteristics and Demographic Representation in Premarketing and Postmarketing Studies of Novel Cancer Therapeutics
Source: JAMA Netw Open. 2021 Apr 20;4(4):e217063. doi: 10.1001/jamanetworkopen.2021.7063 (PMC8058642; doi:10.1001/jamanetworkopen.2021.7063)
Supplement: Supplement. — eTable 1. Inclusion and Exclusion Criteria eTable 2. Novel Therapeutics With Oncologic Approvals by Cancer Type (2012-2016) eTable 3. Availability of Demographic Data by Cancer Type eTable 4. Absolute Difference in Proportion of Women and Older Adults and Proportion by Race Between Study Sample and Patient Population by Cancer Type eFigure 1. Participation to Prevalence Ratio (PPR) for Women by Cancer Type eFigure 2. Participation to Prevalence Ratio (PPR) for Older Adults by Cancer Type eFigure 3. Participation to Prevalence Ratio (PPR) for Race by Cancer Type [file jamanetwopen-e217063-s001.pdf]

Supplemental Online Content

Varma T, Wallach JD, Miller JE, et al. Reporting of study participant demographic characteristics and demographic representation in premarketing and postmarketing studies of novel cancer therapeutics. *JAMA Netw Open*. 2021;4(4):e217063. doi:10.1001/jamanetworkopen.2021.7063

eTable 1. Inclusion and Exclusion Criteria
eTable 2. Novel Therapeutics With Oncologic Approvals by Cancer Type (2012-2016)
eTable 3. Availability of Demographic Data by Cancer Type
eTable 4. Absolute Difference in Proportion of Women and Older Adults and Proportion by Race Between Study Sample and Patient Population by Cancer Type
eFigure 1. Participation to Prevalence Ratio (PPR) for Women by Cancer Type
eFigure 2. Participation to Prevalence Ratio (PPR) for Older Adults by Cancer Type
eFigure 3. Participation to Prevalence Ratio (PPR) for Race by Cancer Type

This supplemental material has been provided by the authors to give readers additional information about their work.

© 2021 Varma T et al. *JAMA Network Open.*

eTable 1. Inclusion and Exclusion Criteria

**Premarketing Studies (Pivotal and Nonpivotal):**	
Inclusion Criteria:	*Exclusion Criteria:*
All studies in the "Review Strategy" section of the FDA medical review documents	Phase 1 clinical trials
	Studies that only evaluated safety of therapeutic
	Studies that only evaluated drug interactions
	Trials with healthy patients
	Pharmacokinetic/Pharmacodynamic (PK/PD) studies
	Bioavailability studies
	Dose escalation studies
	Extension studies
	Expanded access studies
	Studies with patients with a different indication than the indication for which the therapeutic was approved
**Postmarketing Studies (PMRs and PMCs):**	
Inclusion Criteria:	*Exclusion Criteria:*
All PMRs and PMCs indicated in the FDA medical review documents	Non-clinical studies
	Extension studies
	Phase 1 studies
	Studies that were recruiting patients as of July 2020
	PMRs and PMCs that were an aggregate of unspecified studies
	Studies that were listed in more than 1 PMR or PMC

© 2021 Varma T et al. *JAMA Network Open.*

eTable 2. Novel Therapeutics With Oncologic Approvals, by Cancer Type (2012-2016)

**Cancer Types**	**Number of Drugs (%)**	**Drug Name**
Multiple Myeloma	6 (13.3%)	Panobinostat<br>Ixazomib<br>Daratumumab<br>Elotuzumab<br>Pomalidomide<br>Carfilzomib
Leukemias	6 (13.3%)	Venetoclax<br>Obinutuzumab<br>Blintumomab<br>Bosutinib<br>Omacetaxine Mepesuccinate<br>Ponatinib
Melanoma	5 (11.1%)	Cobmetinib<br>Pembrolizumab<br>Nivolumab<br>Dabrafenib<br>Trametinib
Lung Cancer	5 (11.1%)	Osimertinib<br>Alectinib<br>Necitumumab<br>Ceritinib
Breast Cancer	3 (6.7%)	Palbociclib<br>Trastuzumab Emtansine<br>Pertuzumab
Colorectal Cancer	3 (6.7%)	Trifluridine and Tipiracil<br>Regorafenib
Non-Hodgkin's Lymphomas (T-cell, Mantle Cell)	3 (6.7%)	Belinostat<br>Idelalisib<br>Ibrutinib
Ovarian Cancer	2 (4.4%)	Rucaparib<br>Olaparib
Thyroid Cancer	2 (4.4%)	Lenvatinib<br>Cabozantinib
Basal Cell Carcinoma	2 (4.4%)	Sonidegib<br>Vismodegib
Prostate Cancer	2 (4.4%)	Radium-223 Dichloride<br>Enzalutamide
Soft Tissue Sarcoma (including Liposarcoma)	2 (4.4%)	Olaratumab<br>Trabectedin
Urothelial Carcinoma	1 (2.2%)	Atezolizumab
Neuroblastoma	1 (2.2%)	Dinutuximab
Gastric Cancer	1 (2.2%)	Ramucirumab
Renal Cell Carcinoma	1 (2.2%)	Axitinib

© 2021 Varma T et al. *JAMA Network Open.*

eTable 3. Availability of Demographic Data by Cancer Type

Indication	Pre- or Postmarketing Study	Total # of Trials	# of Trials with Sex Data	# of Trials with Age Data	# of Trials with Race Data	# of Trials with Ethnicity Data
Basal Cell Carcinoma	Premarketing	2	2 (100%)	2 (100%)	1 (50%)	1 (50%)
	Postmarketing	2	0 (0%)	0 (0%)	0 (0%)	0 (0%)
Breast Cancer	Premarketing	7	7(100%)	2 (29%)	3 (43%)	1 (14%)
	Postmarketing	6	4 (66%)	4 (66%)	2 (33%)	1 (17%)
Colorectal Cancer	Premarketing	3	3 (100%)	3 (100%)	3 (100%)	1 (33%)
	Postmarketing	1	1 (100%)	1 (100%)	1 (100%)	0 (0%)
Gastric Cancer	Premarketing	1	1 (100%)	1 (100%)	1 (100%)	1 (100%)
	Postmarketing	None	None	None	None	None
Leukemias	Premarketing	8	8 (100%)	7 (88%)	7 (88%)	3 (38%)
	Postmarketing	6	6 (100%)	5 (83%)	5 (83%)	5 (83%)
Lung Cancer	Premarketing	10	10 (100%)	9 (90%)	6 (60%)	6 (60%)
	Postmarketing	4	4 (100%)	3 (75%)	3 (75%)	1 (25%)
Melanoma	Premarketing	7	7 (100%)	7 (100%)	7 (100%)	5 (71%)
	Postmarketing	3	2 (66%)	2 (66%)	1 (33%)	0 (0%)
Multiple Myeloma	Premarketing	11	11 (100%)	10 (91%)	9 (81%)	5 (45%)
	Postmarketing	11	8 (73%)	7 (64%)	5 (45%)	1 (9%)
Neuroblastoma	Premarketing	1	1 (100%)	1 (100%)	1 (100%)	1 (100%)
	Postmarketing	None	None	None	None	None
Non-Hodgkin's Lymphoma	Premarketing	4	4 (100%)	4 (100%)	3 (75%)	3 (75%)
	Postmarketing	9	7 (78%)	7 (78%)	5 (56%)	5 (56%)
Ovarian Cancer	Premarketing	6	6 (100%)	3 (50%)	5 (83%)	0 (0%)
	Postmarketing	3	3 (100%)	1 (33%)	2 (66%)	1 (33%)
Prostate Cancer	Premarketing	6	6 (100%)	3 (50%)	6 (100%)	3 (50%)
	Postmarketing	5	3 (60%)	3 (60%)	1 (20%)	2 (40%)
Renal Cell Carcinoma	Premarketing	4	4 (100%)	3 (75%)	4 (100%)	1 (25%)
	Postmarketing	None	None	None	None	None
Soft Tissue Sarcoma	Premarketing	3	3 (100%)	2 (66%)	3 (100%)	3 (100%)
	Postmarketing	1	1 (100%)	1 (100%)	1 (100%)	1 (100%)
Thyroid Cancer	Premarketing	3	3 (100%)	1 (33%)	2 (66%)	2 (66%)
	Postmarketing	3	1 (33%)	1 (33%)	0 (0%)	0 (0%)
Urothelial Cancer	Premarketing	1	1 (100%)	1 (100%)	1 (100%)	1 (100%)
	Postmarketing	2	2 (100%)	0 (0%)	1 (50%)	1 (50%)
**Total**	**Premarketing**	77	77 (100%)	59 (76.6%)	62 (80.5%)	37[a] (48.1%)
	**Postmarketing**	56	42 (75%)	35 (62.5%)	27 (48.2%)	18[b] (32.1%)

[a]7 premarketing studies categorize ethnicity with race. The categories for race/ethnicity are white, Black, Asian, other and Hispanic.
[b]2 postmarketing studies categorize ethnicity with race. The categories for race/ethnicity are white, Black, Asian, other and Hispanic.

© 2021 Varma T et al. *JAMA Network Open.*

eTable 4. Absolute Difference in Proportion of Women and Older Adults and Proportion by Race Between Study Sample and Patient Population by Cancer Type

Indication	Absolute Difference in the Proportion of Women*		Absolute Difference in Proportion of Older Adults*		Absolute Difference in Proportion of White Patients*		Absolute Difference in Proportion of Black Patients*		Absolute Difference in Proportion of Asian Patients*	
	Premarketing	Postmarketing	Premarketing	Postmarketing	Premarketing	Postmarketing	Premarketing	Postmarketing	Premarketing	Postmarketing
Breast	-0.4	-0.3	-26.8	-21.0	-8.32	-15.2	-7.48	-8.91	11.11	20.09
Colorectal	-6.9	-8.6	-17.7	-19.8	-12.26	-4.02	-10	-10.57	20.52	10.8
Gastric	-8.0	No Study	-24.6	No Study	1.57	No Study	-13.01	No Study	17.48	No Study
Leukemias	-0.6	-0.8	-16.4	-28.5	-4.45	-5.94	-3.83	-3.87	4.59	8.79
Lung	-0.5	13.0	-38.2	-32.5	-19.11	-36.06	-9.47	-9.81	24.28	43.65
Melanoma	-1.6	-3.2	-24.3	-14.7	3.71	2.83	-0.18	-0.48	0.45	0.75
Multiple Myeloma	-1.4	0.9	-12.9	-14.5	8.31	1.9	-14.1	-18.05	3.21	10.32
Neuroblastoma	-6.7	No Study	Not Available	No Study	1.12	No Study	-4.99	No Study	Not Available	No Study
Non-Hodgkin's Lymphoma	-9.4	-11.3	-6.6	-6.6	-5.35	-15.5	-3.43	-5.64	1.87	1.36
Ovarian	0.0	0.0	-9.2	-10.1	2.91	-3.33	-6.91	-8.27	2.23	2.19
Prostate	0.0	0.0	12.8	21.7	15.8	2.39	-3.54	-2.96	-0.29	9.76
Renal Cell	-10.3	No Study	-15.2	No Study	-10.79	No Study	-11.58	No Study	22.54	No Study
Soft Tissue	21.7	13.8	-18.7	-13.4	-3.82	-7.55	-0.51	-9.13	-0.37	15.35
Thyroid	-27.0	-41.9	18.3	0.3	-13.19	No Study	-6.08	No Study	21.09	No Study
Urothelial	-7.6	-7.4	-15.1	No Study	3.42	-15.48	-6.62	-6.25	0.17	11.46

*The absolute difference was calculated as the proportion of women/older adults/white/Black/Asian patients in our sample of studies minus the proportion of women/older adults/white/Black/Asian patients in the U.S. cancer population, by indication. The difference is given in percentage points.

© 2021 Varma T et al. *JAMA Network Open.*

eFigure 1. Participation to Prevalence Ratio (PPR) for Women by Cancer Type

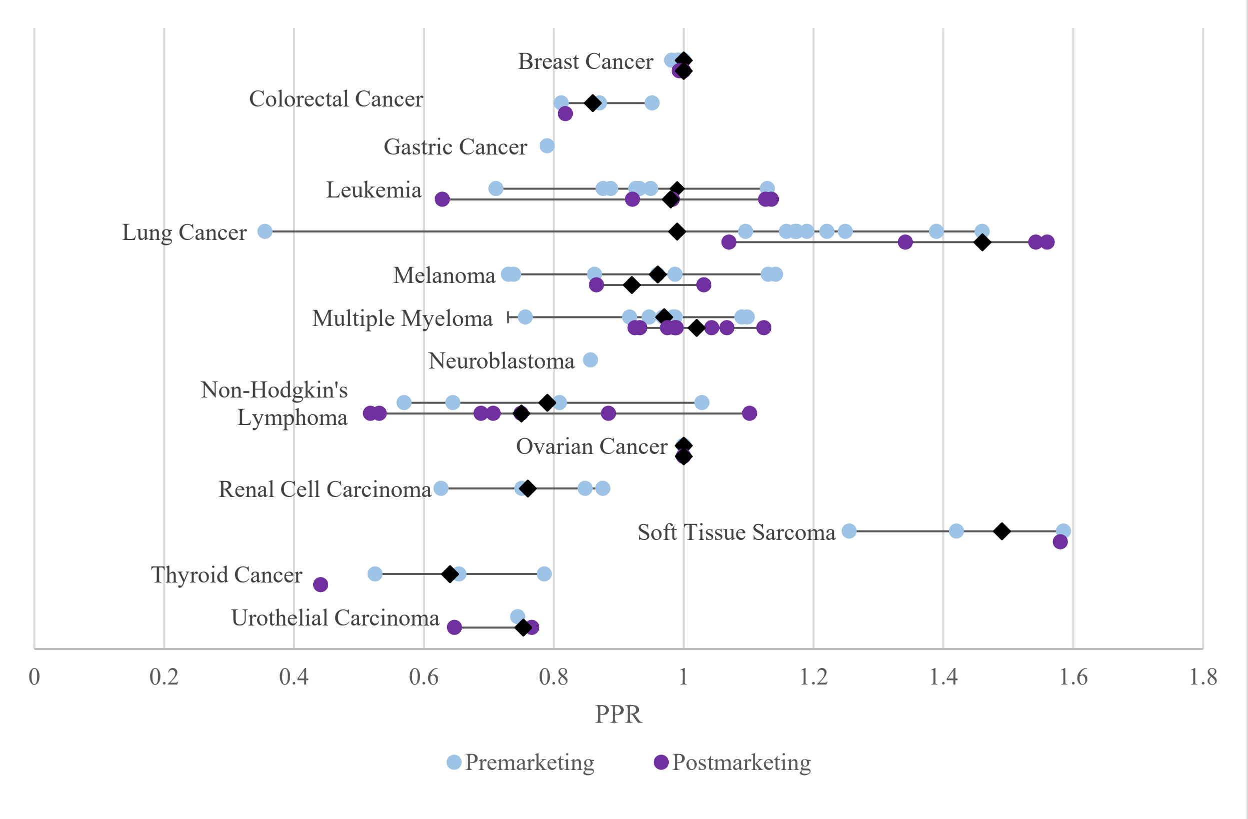

Legend: Each dot indicates the PPR for an individual study (blue dot = premarketing study; purple dot = postmarketing study). The solid black line indicates the range of PPRs (minimum PPR to maximum PPR) for each indication and by premarketing or postmarketing study. The black diamond indicates the average PPR by indication and by premarketing or postmarketing study for indications with more than one premarketing or postmarketing study.

© 2021 Varma T et al. *JAMA Network Open.*

eFigure 2. Participation to Prevalence Ratio (PPR) for Older Adults by Cancer Type

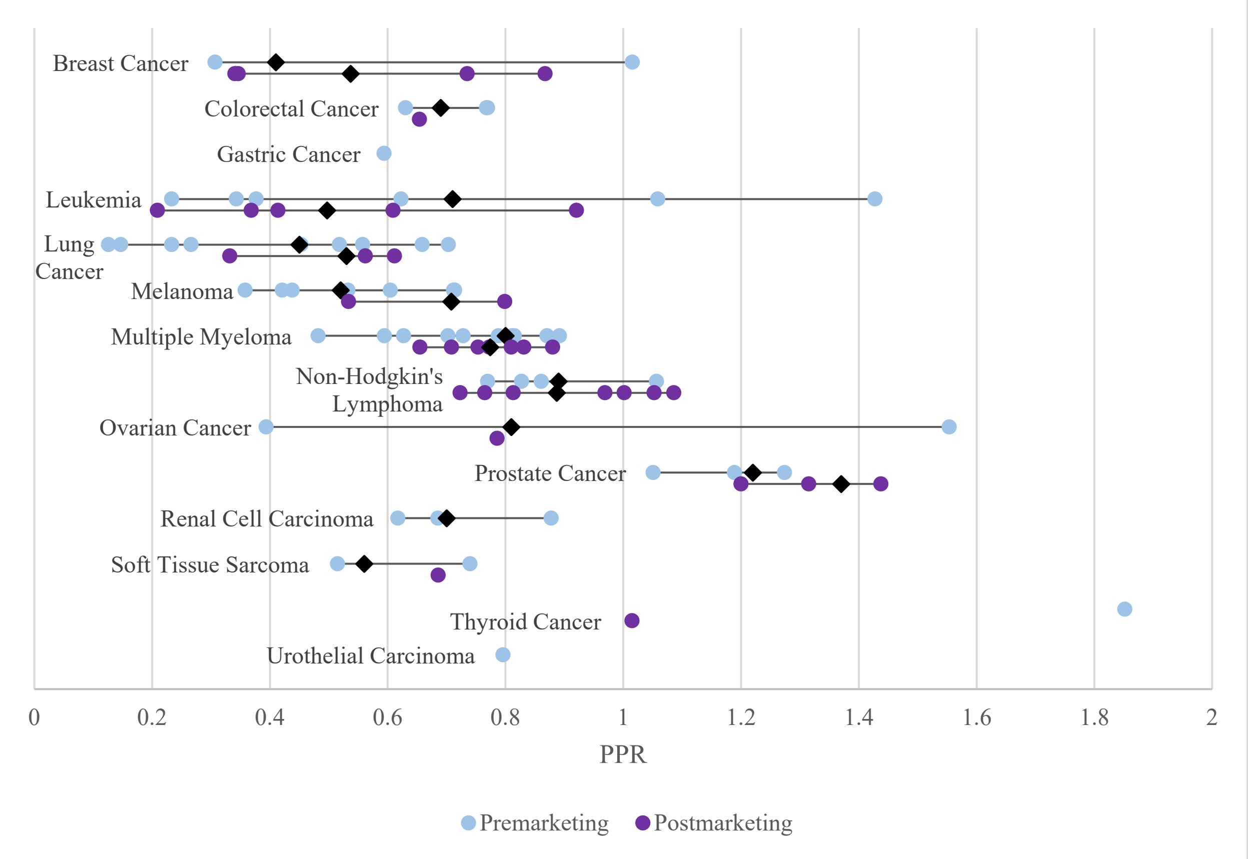

Legend: Each dot indicates the PPR for an individual study (blue dot = premarketing study; purple dot = postmarketing study). The solid black line indicates the range of PPRs (minimum PPR to maximum PPR) for each indication and by premarketing or postmarketing study. The black diamond indicates the average PPR by indication and by premarketing or postmarketing study for indications with more than one premarketing or postmarketing study.

© 2021 Varma T et al. *JAMA Network Open.*

eFigure 3. Participation to Prevalence Ratio (PPR) for Race by Cancer Type

eFigure 3a. PPR for White Patients

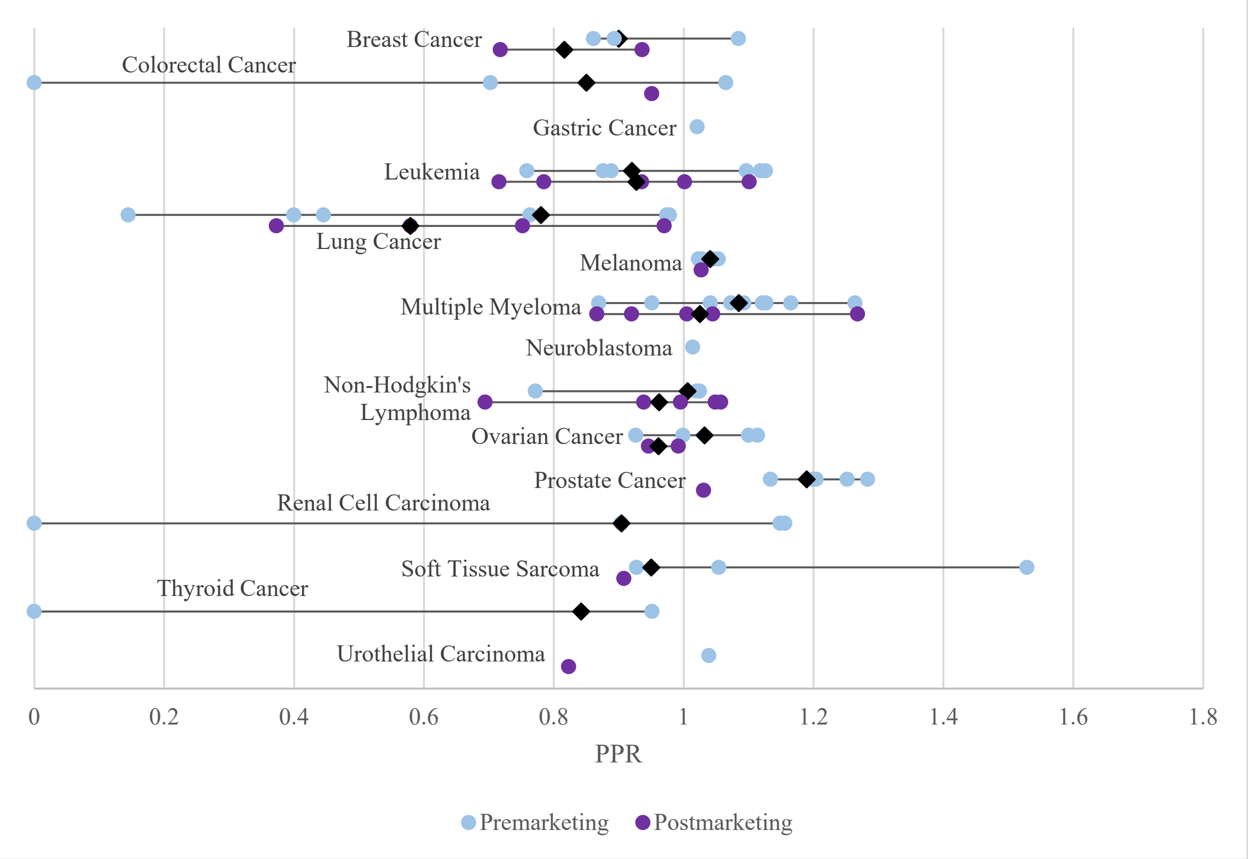

© 2021 Varma T et al. *JAMA Network Open.*

eFigure 3b. PPR for Black Patients

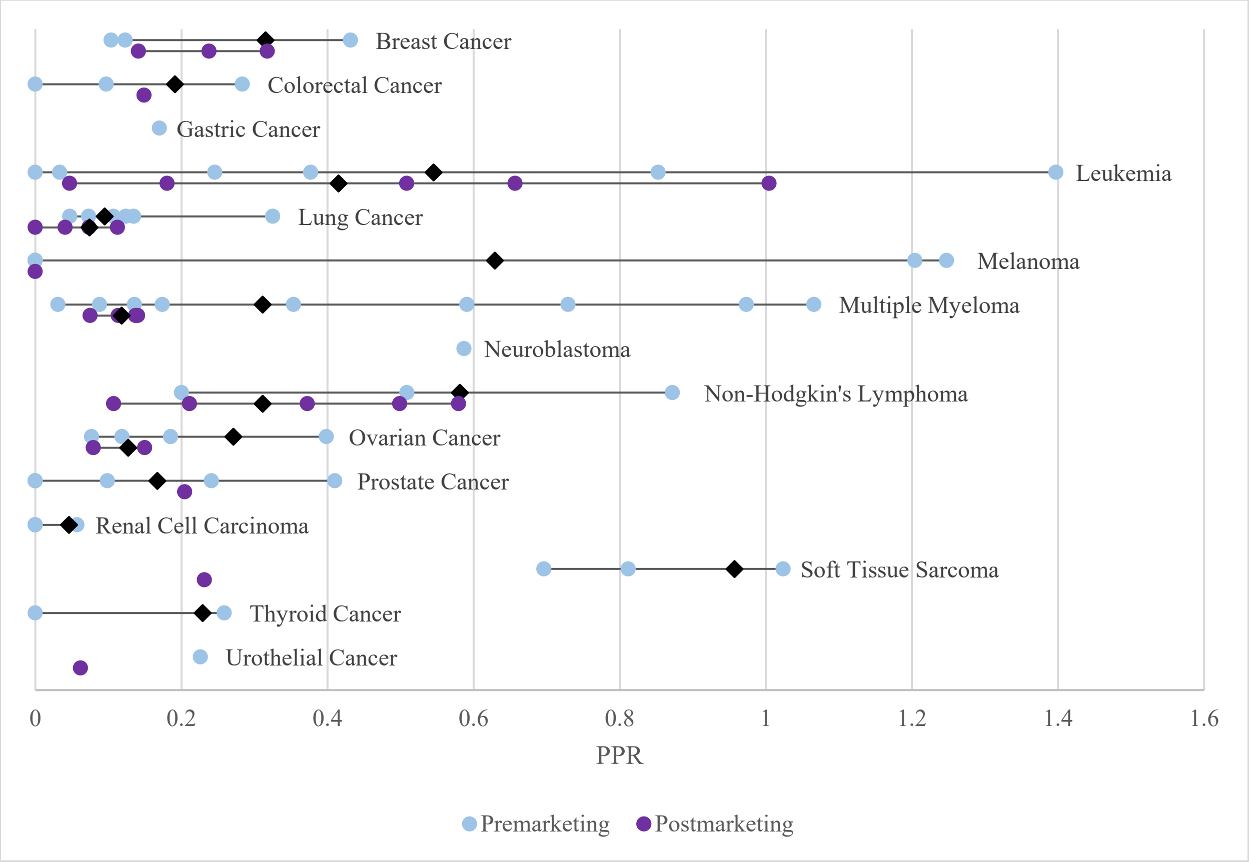

© 2021 Varma T et al. *JAMA Network Open.*

eFigure 3c. PPR for Asian Patients

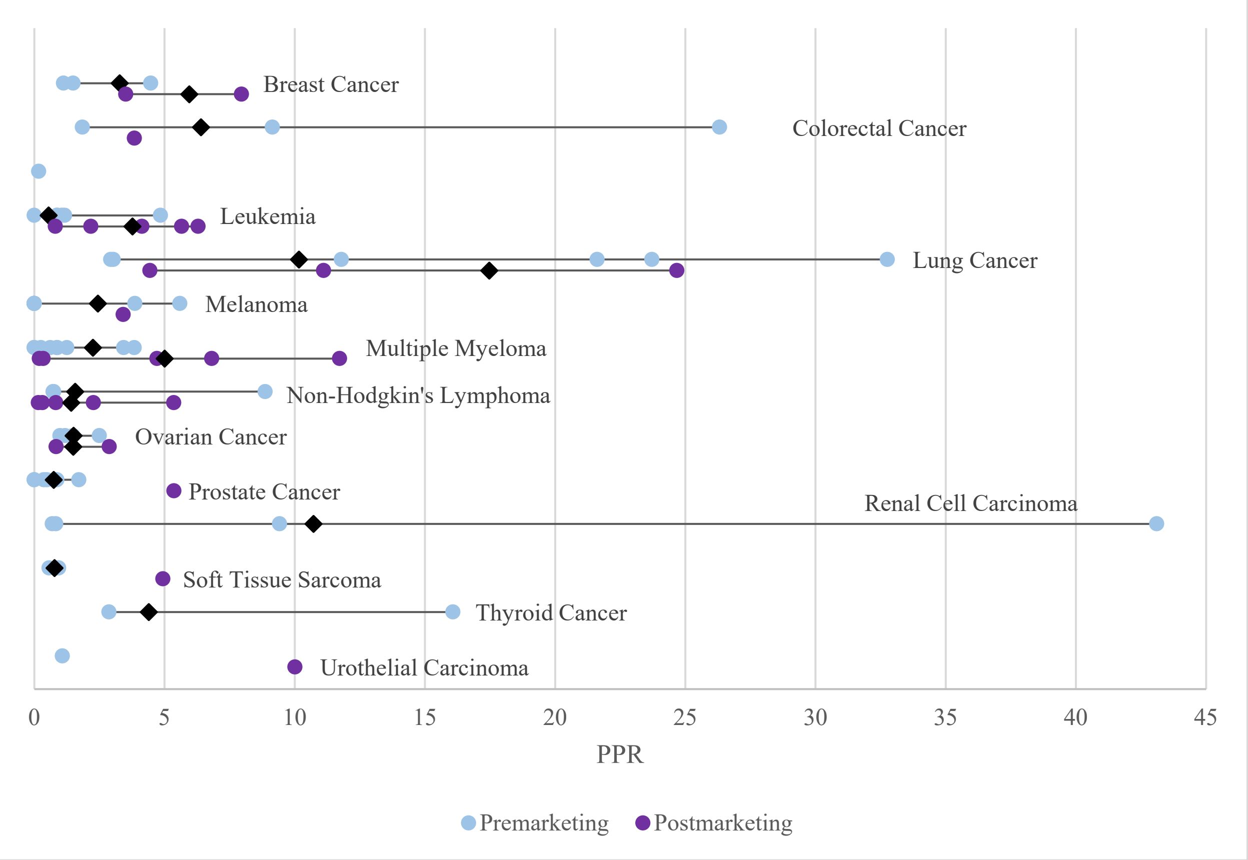

Legend: Each dot indicates the PPR for an individual study (blue dot = premarketing study; purple dot = postmarketing study). The solid black line indicates the range of PPRs (minimum PPR to maximum PPR) for each indication and by premarketing or postmarketing study. The black diamond indicates the average PPR by indication and by premarketing or postmarketing study for indications with more than one premarketing or postmarketing study.

© 2021 Varma T et al. *JAMA Network Open.*
